# Impact of the COVID-19 Pandemic on Disordered Eating Behavior: Qualitative Analysis of Social Media Posts

**DOI:** 10.2196/26011

**Published:** 2021-01-27

**Authors:** Sara K Nutley, Alyssa M Falise, Rebecca Henderson, Vasiliki Apostolou, Carol A Mathews, Catherine W Striley

**Affiliations:** 1 Department of Epidemiology University of Florida Gainesville, FL United States; 2 Department of Anthropology University of Florida Gainesville, FL United States; 3 Department of Psychiatry University of Florida Gainesville, FL United States

**Keywords:** eating disorders, anorexia nervosa, binge eating disorder, COVID-19, coronavirus, Reddit, social media, disorder, eating, qualitative, experience, mental health, theme

## Abstract

**Background:**

A growing body of evidence is suggesting a significant association between the COVID-19 pandemic and population-level mental health. Study findings suggest that individuals with a lifetime history of disordered eating behavior may be negatively affected by COVID-19–related anxiety, and prevention measures may disrupt daily functioning and limit access to treatment. However, data describing the influence of the COVID-19 pandemic on disordered eating behaviors are limited, and most findings focus on individuals in treatment settings.

**Objective:**

The aim of this study is to characterize the experiences of Reddit users worldwide who post in eating disorder (ED)–related discussion forums describing the influence of the COVID-19 pandemic on their overall mental health and disordered eating behavior.

**Methods:**

Data were collected from popular subreddits acknowledging EDs as their primary discussion topic. Unique discussion posts dated from January 1 to May 31, 2020 that referenced the COVID-19 pandemic were extracted and evaluated using inductive, thematic data analysis.

**Results:**

Six primary themes were identified: change in ED symptoms, change in exercise routine, impact of quarantine on daily life, emotional well-being, help-seeking behavior, and associated risks and health outcomes. The majority of users reported that the COVID-19 pandemic and associated public health prevention measures negatively impacted their psychiatric health and contributed to increased disordered eating behaviors. Feelings of isolation, frustration, and anxiety were common. Many individuals used Reddit forums to share personal experiences, seek advice, and offer shared accountability.

**Conclusions:**

Reddit discussion forums have provided a therapeutic community for individuals to share experiences and provide support for peers with ED during a period of increased psychiatric distress. Future research is needed to assess the impact of the COVID-19 pandemic on disordered eating behavior and to evaluate the role of social media discussion forums in mental health treatment, especially during periods of limited treatment access.

## Introduction

The COVID-19 pandemic has disrupted daily living on a global scale, impacting people’s routines, living environments, and physical, mental, and emotional well-being. Many countries have implemented public health measures designed to reduce disease spread, including travel restrictions, quarantine or social distancing policies, and face mask regulations. Media outlets, as well as government and public health officials, are frequently publishing news updates and health recommendations to inform and advise the general public [1]. In response to the stress of ongoing events, individuals may be experiencing unfamiliar or worsening feelings of anxiety, anger, loneliness, and uncertainty. Accordingly, there is increasing concern among health professionals regarding the impact of this pandemic on population-level mental health [2].

Unfortunately, research being conducted during the COVID-19 pandemic indicates that the concern regarding the increase in negative psychiatric symptoms is warranted. In a web-based population survey of more than 1200 respondents from nearly 200 cities in China, the first country to report a cluster of novel coronavirus pneumonia, one in two individuals (53.8%) reported moderate to severe psychological impacts from the pandemic; more than one in four (28.8%) reported moderate to severe symptoms of anxiety, and one in six (16.5%) reported moderate to severe symptoms of depression [3]. Symptoms of anxiety and depression, insomnia, and distress are exacerbated among those particularly vulnerable to COVID-19 exposure, such as frontline health care workers [4,5]. However, those at lower risk for adverse outcomes have also reported increased mental health burden resulting from global prevention measures implemented to reduce COVID-19 transmission. Among Chinese youth, the prevalence of both anxiety and depressive symptoms during home confinement exceeds prevalence rates published in studies prior to the COVID-19 pandemic [6]. In the same sample, low optimism regarding the epidemic was associated with increased risk of depressive symptoms.

In addition to increasing symptoms of depression and anxiety, there is concern that the negative emotional effects of the pandemic and associated public health measures will exacerbate eating disorder (ED) triggers and symptoms. Qualitative exploration of ED patients conducted during the initial stages of home confinement supports this presumption; of 32 recovering patients, nearly all expressed concern regarding the negative influence of increased uncertainty related to the pandemic on their daily lives and treatment progress [7]. More than one-third of surveyed patients reported exacerbation of ED symptomatology, and over half reported an increase in anxiety-related symptoms. Large population-based studies have observed similar changes in disordered eating behaviors during the pandemic among people with and without ED. Indeed, of 180 Australian respondents with a self-reported ED history, two in three reported increased restriction, one in three reported increased binge-eating, one in five reported purging more frequently, and nearly half reported increased exercise behavior [8]. Although the majority of people without ED (n=5289) reported no change in disordered eating behaviors, nearly one-third (28%) reported increased food restriction and 35% reported an increase in binge-eating. Notably, for a large number of individuals with current or past ED, living environment, daily routine, and positive coping behavior has been negatively impacted by COVID-19 prevention measures, resulting in a decreased sense of control and the worsening of ED symptoms [9-11]. In contrast, as confinement measures are gradually reduced, significant decreases in ED symptomatology and emotional dysregulation have been observed [12].

Rodgers and colleagues [13] have proposed three distinct pathways through which the COVID-19 pandemic may worsen ED risk. First, it is predicted that public health prevention measures used to decrease COVID-19 spread will adversely affect access to care and social support networks needed for people with ED symptomology. While the medical community has focused its attention on managing the spread of COVID-19, the already difficult challenge of reaching individuals with ED may worsen, treatment-seeking and diagnosis may be delayed, and treatment uptake and engagement may be less effective [14,15]. For many patients receiving psychiatric care, consultation and treatment via telemedicine have become commonplace in recent months [16,17]. Some clinicians have expressed concerns regarding the effectiveness of web-based therapy, especially among those unfamiliar with telehealth [18]. Similarly, despite evidence that most ED patients express favorable attitudes toward teletherapy [14,19], some may question treatment quality or view telehealth as “second best,” and willingness to engage in treatment may be reduced [18]. In two separate investigations related to the telehealth transition, ED patients reported feeling like a burden or an inconvenience following dismissal from or suspension of treatment due to the COVID-19 pandemic [10,11]. Some individuals further endorsed the belief that telehealth was not equivalent to in-person treatment [10,11] and reported emotional distress and negative body image stemming from the video requirements of telehealth [10]. Additionally, acceptance of telemedicine may vary by ED subtype, with evidence suggesting that individuals with the anorexia nervosa subtype may be least content with the transition to remote treatment [12]. In inpatient settings, care teams have been reduced in size, visitations have been limited, and admission criteria have become more stringent [16,17,20]. To abide by social distancing guidelines, group therapy sessions have been cancelled or conducted remotely, limiting patients’ access to familiar social support networks, though also reducing opportunity for body comparisons [20]. Additionally, physical and financial barriers to treatment may arise among patients with limited computer or internet access [18] and among those whose income has been adversely affected by business closures. For some, web-based ED information and self-help materials may serve as important resources during the pandemic [14]. However, it is noted that high-quality and easy-to-comprehend web-based content may be difficult to obtain [14,21].

Second, it is possible that people who have or are at risk of having an ED may experience increased exposure to ED-specific media or media which increases anxiety related to food, exercise, and weight [13]. In recent months, researchers have identified more than 15,000 Instagram posts referencing the “quarantine-15,” a phrase that mirrors the more common “freshman 15,” which is often used to describe weight gain among first-year college students [22]. Some social media posts display indulgent foods that may trigger binge eating, and other posts imply that it is vital to avoid increased body weight by exhibiting weight-stigmatizing content and negative characteristics stereotypically associated with obesity, such as laziness and lack of self-control [22]. In combination with increased media consumption during periods of social distancing, it is likely that greater attention to weight- and food-related content may trigger or exacerbate ED symptoms, given the negative influence of the thin ideal frequently romanticized on social media [23]. In one study, more than half of participants with ED reported the worsening of ED symptoms following increased exposure to food and exercise social media content since implementation of public health prevention and lockdown measures [10]. Although some ED patients, particularly those in recovery, may modify social media accounts to turn attention to positive or recovery-focused content [7], those with untreated ED and those at risk for ED may be especially harmed by these media trends. Across existing studies, ED individuals consistently reported anxiety related to physical activity and the inability to exercise [9-11,24], especially after exposure to exercise-related media content [10].

Third, fear of COVID-19 infection may increase anxiety over food quality, quantity, and potential transmission via certain food products. Specifically, Rodgers and colleagues [13] hypothesize that restriction may occur via reduced purchasing of specific food items and reluctance to leave home to purchase groceries given one’s fear of contagion. A positive association between fear of COVID-19, eating restraint, and concerns related to weight or shape emerged from an investigation among both the general population and individuals attending diet clinics for weight loss management [25]. Grocery shopping during the pandemic may prove difficult for those with disordered eating. Touyz and colleagues [26] postulate that problematic relationships with food, including both restriction and binge eating may be exacerbated by food shortages and panic buying. Rigid and inflexible eating behaviors may be challenged by purchasing restrictions and low supply of certain products or brands deemed “safe” by those with restrictive EDs. In one study of 1021 individuals with lifetime ED, more than two-thirds reported being slightly or very concerned about accessing foods consistent with their current meal plan or style of eating [9]. At the same time, food hoarding and the inability to distance oneself from food at home may prove challenging to those with binge eating disorder [15,26,27]. If food is shared among members of the household, bingeing on the family’s food supply may introduce unnecessary family conflict [15,26]. Furthermore, as pandemic-related economic strains spread worldwide, financial hardship may give rise to food insecurity—a known correlate of binge-eating behavior [14,23].

Although a number of researchers have expressed similar concerns regarding the impact of the pandemic on disordered eating behaviors, data supporting these hypotheses are limited. Accordingly, this qualitative analysis characterizes the anonymous experiences of Reddit users posting original content in ED-related discussion forums, in which they describe the ways that the COVID-19 pandemic has influenced their mental health and engagement in disordered eating behaviors.

## Methods

### Data Collection

Reddit, an internet-based social media platform consisting of news stories, web content ratings, and discussion communities, has been denoted as a valuable tool for collection of high-quality psychological research data [28]. This web-based forum is divided into communities of registered users expressing interests in unique discussion topics (ie, subreddits). In this analysis, data were collected from three popular subreddits that acknowledge EDs as their primary discussion topic: r/EatingDisorders (43,500 members), r/AnorexiaNervosa (19,200 members), and r/BingeEatingDisorder (35,700 members). Unique discussion posts dated from January 1 to May 31, 2020, that referenced the COVID-19 pandemic (ie, containing one or more of the keywords *coronavirus*, *COVID*, *quarantine*, or *pandemic*) were extracted using the R RedditExtractoR package (R Project). In addition to the title and content of the public posts, the username of the posting entity, posting date, and number of reply comments were collected. Only the initial posts, and not subsequent replies, were included for data analysis. All discussion posts were written in the English language; thus, language-based exclusions were not applied. As this secondary data analysis is limited to publicly available, web-based content, this research was determined by the University of Florida’s Institutional Review Board to be exempt from human subject review. Although the discussion threads are publicly available, usernames have been omitted from this report to protect user anonymity.

### Statistical Analysis

Inductive, thematic data analysis was used to elucidate patterns in the data and construct themes describing thoughts and behaviors common to users of ED subreddits during the COVID-19 pandemic [29]. All posts were initially reviewed by one member of the research team (SN). The researcher coded posts line-by-line to gain familiarity with the data and to identify commonalities between threads. Posts representative of multiple thematic constructs were coded into multiple categories as applicable. The researcher (SN) independently developed an initial codebook to outline the scope of topics discussed by users. Two-thirds of the discussion posts (206/305, 67.5%) were then randomly selected to be coded by two additional members of the research team (AF and RH; ie, one-third to each member) to determine disagreement and to finalize codes. Similar codes were grouped into common categories, which were used to construct overarching themes. These themes and associated codes were discussed as appropriate to reach consensus. If consensus was not achieved, a third member of the research team (VS, CS) was consulted for review of discrepant items, and consensus was reached between the three coders. A clinician was available to provide expertise as needed (CM). Once all discrepancies were discussed and agreement was met, a final copy of the codebook was developed (Multimedia Appendix 1) and applied to the data for final code frequency reporting.

## Results

### Characteristics of Discussion Posts

In total, we identified 33 relevant posts from 33 unique users in r/AnorexiaNervosa, 180 posts from 172 users in r/BingeEatingDisorder, and 92 relevant posts from r/EatingDisorders (restricted subreddit: username and count unknown) that were published between January 1 and May 31, 2020. Collectively, the posts consisted of 66,877 words. The posts were approximately 215 words in length and received between 1 and 73 replies (mean number of comments: 6 [SD 7.7]).

### Themes

Six primary themes were identified: change in ED symptoms, change in exercise routine, impact of quarantine on daily life, emotional well-being, help-seeking behavior, and associated risks and health outcomes. Primary themes comprised subordinate themes (secondary themes), as detailed in Table S1 in Multimedia Appendix 1.

### Change in ED Symptoms

Content that fell under this overarching theme was specifically related to disordered eating behavior and thought patterns that were influenced by the COVID-19 pandemic and the corresponding preventative measures.

#### Increased ED Symptomatology

Users often experienced a worsening of ED symptoms corresponding to the onset of the global COVID-19 pandemic. With limited opportunity to leave the home or engage in personal and social activities as a result of quarantine and social distancing guidelines, in two-thirds of posts (201/305, 65.9%), users struggled to ignore obsessive thoughts related to food and weight and to refrain from engaging in disordered eating behaviors prompted by loneliness, anxiety, and boredom. For example, users with self-reported binge eating disorder often described quarantining at home as being “surrounded by food” and described “feeling as if [they wouldn’t] be able to control [themselves] around all the food.” Additionally, many users with ongoing ED symptoms relied on increased frequency of compensatory behaviors (ie, restriction, purging) to cope with changes in their day-to-day routine introduced by the pandemic. One user wrote*,“*I feel like since I'm not going to be walking and active as much, I need to be even more ‘diligent’ with calorie/counting, etc.”

Some users who described themselves as recovered or in recovery sought comfort in a time of heighted emotional distress by engaging in ED behaviors previously associated with feelings of comfort and control. These users found the newly implemented COVID-19 restrictions to be very disruptive to the recovery mindset and other recovery-oriented practices. One user who described themselves as currently in recovery stated:

Quarantine has given me too much time to think about my loss of control and all of the negatives I've held against myself over the years. […] As the days go on, I feel myself slipping into loneliness, depression, and anorectic tendencies.

For a small number of individuals, disordered eating behavior was not present prior to the pandemic. These users primarily sought to gauge the severity of their diet behavior and their newfound anxieties related to food and weight:

With this quarantine going on and everyone in my country are required to stay at home, I didn't get the chance to exercise. I lost control, ate a lot of snacks and every time I weigh myself I see the numbers fluctuating. […] I started to restrict even more, I stopped having my dinners and I purchased weeks worth [sic] of meal replacements. I just wanted to get to the weight I at least liked myself in.

#### Decreased ED Symptomatology

One in ten users (n=37, 12.1%) reported less frequent or less severe ED symptoms corresponding to the onset of the COVID-19 outbreak. These users primarily discussed using the pandemic as an opportunity to focus on ED recovery and had successfully reduced the frequency or severity of disordered eating behaviors. However, many of those with decreased ED symptomatology expressed concern regarding reversion to ED behavior, as COVID-19 prevention measures were lifted. For one user who experienced a decrease in ED behavior while in quarantine, this concern was particularly relevant:

Since the covid started I tried to focus on my eating disorder and bringing myself to have a healthier relationship with food. I thought I was doing pretty good, because my mom was home and she was the one that made meals and pushed me to eat. This week she has gone back to work, and I’ve sort of been struggling a lot. I struggle with depression so it’s just really hard to bring myself to make a meal.

#### Negative Body Image

In approximately one-fifth of posts (64/305, 21.0%), users reported having more time to scrutinize their bodies and eating behaviors. An increase in body-checking behavior while confined at home was frequently discussed. For some, body dissatisfaction negatively affected daily functioning. One user wrote:

I’m scared to look in a mirror, and taking a shower sounds practically terrifying at this point. My body dysmorphia is killing me, much to the point where I can’t even get up to change clothes.

For a large majority of users reporting negative body image, discomfort with subjective changes in body weight or shape led to increased frequency or severity of compensatory behaviors, including restriction, overexercising, vomiting, and laxative use. One user posted,

With the recent quarantine, I am unable to work out the same… just running and doing as much as I can. I’ve found my body changing in ways I am very uncomfortable with. I’m waking up daily weighing myself, logging my food, and checking my Fitbit constantly. I recently started staring in the mirror more and despising the person who looks back.

### Exercise Routine

Content that fell under this overarching theme was specifically related to the influence of COVID-19 on exercise behavior.

#### Change in Exercise Behavior

Users who engaged in regular exercise often reported difficulty maintaining their exercise routine (41/305 posts, 13.4%). While some users reported decreased motivation to engage in regular exercise, others reported difficulty avoiding excessive exercise. One user described their need to engage in additional exercise to “earn” food—a habit that the user described as both exhausting and anxiety-inducing.

*Since the beginning of my forced social isolation, exercise has ramped up to now four hours a day of walking […] It has become consuming and I can't stop no matter what I do. […]* *Please, please someone help me.*

Changes in exercise behaviors had large ramifications for appetite that many users struggled to cope with. Some users aiming to increase physical activity reported discomfort responding to increased appetite. For some, this internal struggle resulted in binge eating behavior, which then tempted them to exercise excessively.

#### Exercise Facilities Closed or Inaccessible

A small portion of users who engaged in regular exercise expressed frustration with exercise facility closure mandates (13/305 posts, 4.3%). Users explained that their exercise routine served as an alternative to dangerous purging behaviors (eg, vomiting, laxative use) and often helped them manage their ED thoughts and behaviors. Many of those who lost access to exercise facilities as a result of COVID-19 closures experienced difficulty adjusting their exercise routines and reported significant loss in motivation to exercise. Emotional distress and feelings of guilt were common, with a number of users communicating fears of “falling into old habits” without access to the gym.

#### Impact of Quarantine on Daily Life

Content that fell under this overarching theme described the influence of COVID-19 restrictions on participants’ daily routines, living environments, purchasing behaviors, and interpersonal relationships.

#### Change in Routine/Environment

More than one-third of users (119/305 posts, 39.0%) discussed the ways in which COVID-19 prevention measures directly disrupted their everyday routine. Unable to attend work, school, and social gatherings, many users grappled with boredom and loneliness. As a result of business closures, a small number of users experienced job loss. These individuals felt very defeated and reported experiencing obsessive thoughts related to food and body weight while trying to avoid engaging in ED behaviors.

Some individuals were frustrated by the routines of their friends and family members. Being in close proximity to friends and family members for an extended period of time, some users described a loss of privacy. Some users reported that their family members were unaware of their disordered eating behavior and reported feeling anxious or frustrated when they were unable to engage in compensatory behavior. Other users reported being negatively influenced by the coping behaviors of their family and friends with whom they were quarantining. For example, one user shared,

I have been quarantined with my grandmother for the past 3 weeks and will continue to be quarantine with just her for the next month. She keeps cooking out of boredom and trying to hand me giant plates of food every few hours. […] Her CONSTANTLY making me food is so beyond triggering I can not [sic] handle being around it anymore.

In terms of environment, some users were required to relocate to an alternate living space due to quarantine and social distancing guidelines. For example, a number of college-aged users discussed the need to return to their parents’ homes as a result of university closures. For some, this change resulted in the loss of social networks guiding recovery efforts. One user wrote,

My college is closed for the remainder of the semester and with that went my support system and all of my friends. School is where I have made the most progress in terms of recovery because I had my boyfriend and friend provided nothing but positivity and support. However, I can’t say that’s the same at home.

Users in similar situations anticipated that the home environment would increase personal anxieties and trigger ED behaviors. Many communicated fear of relapse, with one user discussing their fear of reverting to ED behavior while “trapped with those who only make things worse for [them].” In addition to personal relocation, some users experienced changes within their environment, such as roommate travel or relocation.

#### Food Hoarding or Shortages

Some users experienced significant distress related to changed food availability (34/305 posts, 11.1%). In preparation for citywide or nationwide quarantine mandates, users reported pressure to purchase nonperishable food items in excess of their immediate needs. For these users, the stockpiling of food items, especially those deemed “binge-worthy,” instigated intense negative reactions and compulsive urges to act on disordered thoughts and feelings. One user wrote,

As a binge eater, it is really hard to grocery shop right now. I know I should go in with a list/plan. But it’s like, oh should I get pasta? That will keep for any quarantine. But I’m probably gonna eat a box tonight, so should I get 5 boxes to stockpile? Well then I’ll eat a box of pasta every night… then it’s like, well might as well get some ice cream and chocolate cuz I already [expletive] up and am eating a box of pasta tonight.

When the supply of nonperishables was meant to serve the entire household, users often grappled with the guilt they associated with diminishing the family’s stockpile. Many felt they were “wasting” food and worried that household conflict would arise if family members knew they were binge-eating or purging the “COVID food.”

Other users responded negatively to the limited food supply available in grocery stores and supermarkets. As the supply of “safe” or routine foods dwindled, users expressed concern that they would be unable to find foods they felt comfortable eating, or that they would be able to maintain the meal plan administered by their treatment provider. Similarly, some users expressed frustration with the inability to binge eat as a result of restaurant closures and reduced availability of “binge” or “trigger” foods in grocery stores and supermarkets.

#### Navigating Triggering Relationships

As a result of quarantine restrictions, users sometimes faced more frequent interaction with friends or family members who instigated feelings of anxiety, shame, or loss of control (45/305 posts, 14.8%). Unable to eat in private or avoid family meals, many users expressed feelings of sadness and anger after being subjected to criticism regarding their eating behaviors. A large number of individuals reported that their family members did not understand their relationship with food and often felt that their concerns were easily dismissed or ignored by family members. One user explained,

My mom is taking my anxiety and depression as a joke and I don’t know what else to do. I’ve tried talking to her about how I’m having harmful thoughts and how I can’t stand eating all this food and not going to work to work any of it off and how I’m starting to gain weight… what does she do she calls her friend and is laughing with her friend because “oh she being dramatic and just has cabin fever” like what? No! It’s not something that should be taken as a joke.

Users frequently experienced disordered thoughts after being “forced to eat something” by parents or family members, especially if the meal was deemed a “forbidden” or “triggering” food. For many, body dysmorphia and fear of weight gain was reinforced by increased consumption. Negative body image was further triggered by family members who criticized the body weight or shape of users. One user stated,

My mom had always joked about my body/weight (since I used to be overweight, she would joke about me not being able to fit through my door). She don't mean any harm, but I get really devastated when she mentioned that I've gained weight during quarantine. I started to restrict even more.

### Emotional Well-Being

Content that fell under this overarching theme specifically referenced users’ moods and emotional health.

#### Negative Affect

In nearly two-thirds of the posts (191/305, 62.6%) users reported that they experienced negative emotions in response to current events or disordered thoughts and behaviors. Commonly endorsed emotions included fear or anxiety, loneliness and isolation, anger or frustration, guilt or shame, and hopelessness or depression. A large number of users had difficulty reasoning through their emotions. For example, one user wrote,

all I’ve been doing is just lying around. i love movies, but i can’t even bring myself to watch a lot for some reason, I’m just really sad. as for my ED, i keep struggling with the same relapses over and over again […] i feel disgusting! i don’t know what to do at this point, i feel so alone.

In general, users felt very defeated by negative emotions. Many users sought support from the Reddit community; others stated they did not seek guidance, but rather a means through which they could convey their thoughts and feelings without judgment.

### Help-Seeking Behavior

Content that fell under this overarching theme detailed users’ attempts to seek professional or informal help for disordered eating behavior during the COVID-19 pandemic.

#### Willingness to Recover

In 78/305 posts (25.6%), users communicated a strong willingness to use quarantine mandates as an opportunity to focus on eating disorder recovery. These individuals felt that the absence of a daily routine allowed additional time to curb addictive habits and develop healthier habits. One user described two options for his or herself during quarantine:

1. I continue to binge eat and worsen my habits out of boredom and depression.

…

2. I use these 2-4 (who knows how long) months to finally adopt the habits I’ve yearned for and become the person I want to be. I eat right and exercise. I have small daily goals that I accomplish to keep myself distracted.

Introspection of disordered behavior was common among those aiming to reduce eating disorder symptoms. Many users discussed the identification of triggering food, people, environments, and media. Some provided a short plan to avoid such triggers. Some provided other subreddit members with progress updates and celebrated recovery-focused accomplishments.

#### Currently Receiving Treatment

Approximately one-tenth of users (30/305 posts, 9.8%) mentioned participating in ongoing treatment programs. However, a small number of these individuals reported discontent with their provider, felt that their provider did not understand the severity of their disorder, or reported that their treating professional did not specialize in eating disorders. Some users expressed concern that telemedicine and internet-based treatment were not sufficient for the level of care they required. One user described treatment during COVID-19 as lacking sufficient structure and support necessary to stifle disordered thoughts and behaviors.

#### Unable to Receive Treatment

In approximately one-tenth of posts included in this analysis (31/305, 10.2%), users reported the inability to receive ED treatment as a result of the COVID-19 pandemic. For some, this entailed disruption to their ongoing treatment plan or treatment schedule. A few users were negatively influenced by the sudden loss of access to higher-level care. One user posted,

I have been mentally preparing myself for residential treatment […]. Today I found out that my move in date is cancelled until further notice due to covid-19. Trying to navigate this disorder everyday is exhausting and now I have to keep trying to do it at home. It has me feeling completely hopeless and defeated.

Other users lost access to treatment conducted in the group setting, which left them feeling increasingly isolated. In general, users felt that it had become increasingly difficult to remain in contact with their treatment providers.

Those not currently in a treatment program often reported that traditional barriers to care, such as fees and waiting lists, were exasperated by the ongoing medical crisis. These individuals reported feeling hopeless and often sought advice and support from other Reddit users in lieu of treatment.

#### Requesting Advice or Accountability From Other Reddit Users

In many posts (152/305, 50.2%), users called for support or guidance from others experiencing similar thoughts and behaviors. The request for an “accountability buddy,” a fellow user committed to helping another user confront challenges during recovery efforts, was common. Some users requested support in unraveling difficult emotions and responding to challenging or triggering persons or environments.

#### Words of Encouragement

In 29/305 posts (9.5%), individuals aimed to encourage and uplift others by discouraging disordered thoughts and behaviors and reaffirming users’ ability to recover and develop normalized eating patterns. Most users described their own experiences with ED before offering support to other users. Some discussed their own recent victories and offered motivational comradery, often by reminding one another that they were not alone. One user wrote,

I just wanted to let those of you guys really struggling mentally right now that I’m right here with you and that we’re gonna make it through this.

In some cases, users appeared to provide words of self-encouragement using journal-style entries. Prior to journaling food consumption for the day, one user recorded their personal mantras, which included statements such as “It is worth it” and “It will get better.”

#### Seeking Help on Behalf of Another Individual

Ten users (3.3%) posted in search of advice of how to help a loved one with an eating disorder as they dealt with pandemic-related anxiety. These users reported noticing their family member or friend engaging in ED behavior for the first time or to a greater extent than what was observed prior to the pandemic. Users struggled to find ways to support those with ED without encouraging ED behavior. One user wrote,

My GF and I started dating about 2 weeks before she went to treatment for Ana. […] This Covid-19 scare has her wrecked. […] How can someone be supportive, but not be enabling?

Many of these individuals also provided words of encouragement to those with ED. One user stated,

Be compassionate and patient with yourself! I don’t want to lecture anyone of ED’s when I don’t have one myself, but my heart goes out to anyone reading this. Anyway, good luck peeps and keep up the good work!

### Associated Risks and Outcomes

Content that fell under the final overarching theme described adverse health behaviors and outcomes that individuals with ED grappled with during the pandemic.

#### Substance Use Behavior

A small number of users (20/305 posts, 6.6%) described using psychotropic medications to alleviate symptoms of depression or anxiety. Some of these users reported misusing these medications in an effort to suppress weight gain. Other reported using cigarettes, Adderall, or laxatives in an effort to suppress one’s appetite or lose weight. Some users were recently prescribed or were currently seeking a prescription for weight loss medication. Most of these individuals were hopeful that weight loss medication would reduce the urge to binge-eat.

#### Adverse Health Outcomes

In approximately 1 in 10 posts (31/305, 10.1%), users reported experiencing physical pain, gastrointestinal distress, dental distress, or dehydration symptoms as a result of disordered eating behavior. Many felt ashamed of the associated health outcomes and were reluctant to notify others of their physical distress or seek medical attention. Only one user reported that experiencing adverse health events would increase willingness to reduce disordered eating behavior. A small number of individuals expressed concern that their ED (and associated health conditions) would increase their vulnerability to COVID-19.

## Discussion

### Principal Findings

The objective of this analysis was to characterize the impact of the COVID-19 pandemic on disordered eating behavior using inductive, thematic analysis of ED-related Reddit forums. Consistent with previous investigations [7-12,24,25,30], our findings indicate that the COVID-19 pandemic and associated public health prevention measures negatively impacted the psychiatric health of most users. Users frequently sought comfort in a time of heighted emotional distress by engaging in ED behavior, which was exacerbated by abrupt and involuntary changes in daily routine, exposure to food shortages and panic buying, closure of exercise facilities, and reduced access to care and social support networks. Feelings of isolation, frustration, and anxiety were common, and users frequently sought advice or encouragement from other Reddit users to overcome negative thought patterns. Despite negative emotionality, a small number of users reported using the quarantine mandate as an opportunity to focus on ED recovery and reported successfully decreasing ED symptomatology since the onset of the pandemic.

The findings of this analysis partially align with the hypotheses of Rodgers and colleagues [13], which posit that the COVID-19 pandemic worsens ED risk via limited access to care and social support networks, increased exposure to ED-specific media, and anxiety related to changing food quantity and quality. Reddit users included in this analysis frequently discussed the disruption to or cessation of ED treatment following the onset of the pandemic and struggled with limited access to both formal and informal support networks—a finding that supports the first hypothesis of Rodgers and colleagues [13] and expands on findings from a similar qualitative investigation conducted using a smaller sample of adults with self-reported ED [31]. Our findings also supported the third hypothesis of Rodgers and colleagues and suggested that changed food availability contributed to increased anxiety and ED behavior. Although fear of contagion did not appear to increase food-related anxieties, low supply of certain food products deemed “safe” by those with restrictive ED increased feelings of fear and frustration. Additionally, home stockpiling of food items instigated intense negative reactions and compulsive urges among those engaging in binge-eating and purging behaviors. However, our findings do not support the second hypothesis of Rodgers and colleagues, who postulate that increased exposure to ED-specific media during the COVID-19 pandemic increases risk of ED behavior. In part, this may be the result of data collection via social media.

Notably, this analysis found that peer-based encouragement and role modeling of positive behavior was also common among users of ED subreddits, which may be expected of a social media forum. Users eager to reduce ED behavior shared encouraging stories and messages of support and offered one another shared accountability. In this way, our findings indicate that Reddit forums may function as a positive, therapeutic community for individuals with ED symptoms during periods of heighted emotional distress. This may be especially important during the COVID-19 pandemic, given users’ reports of limited access to professional psychiatric care, ED group therapy, and informal social support networks. However, there is evidence that all ED Reddit forums, even those specifically promoting ED recovery, should be carefully moderated to minimize exposure to harmful content promoting ED behavior and to maximize effectiveness as a helpful platform for individuals with ED [30]. Thus, our findings add to the growing body of research that considers the potential role of health care providers in web-based discussion forums. Certainly, health providers treating individuals with ED would benefit from increased understanding of the unique challenges common to those with ED during the pandemic. Therefore, there may be an emerging role for clinicians to monitor such forums as a means to understand the effects of important events such as the COVID-19 pandemic. Additionally, individuals with ED, especially younger patients, may find value in patient-provider communication through such venues, which may be considered more accessible and less anxiety-provoking [32]. However, ethical concerns remain (ie, privacy concerns, professionalism, quality of information), and clinicians have no way to be remunerated for such a role. As social media grows as an integral form of communication between health providers and their patients, it has been recommended that medical professionals receive education to effectively communicate with users and patients via the internet, such that the spread of misinformation is minimized and quality of care is maximized [33]. This may be especially important for ED, as high-quality and easy-to-comprehend internet-based information related to ED is often difficult to obtain [14,21]. In addition to ethical concerns, there is no guarantee that those who use Reddit would be open to clinical guidance via discussion forums; such posts could divert the Reddit platform from its established social function. While acknowledging these concerns, further research should address the acceptability, availability, and effectiveness of clinician participation in such forums.

### Limitations

Our findings should be considered in the context of our study’s limitations. First, it is possible that additional Reddit discussions related to eating disorder behavior and the COVID-19 pandemic were not considered in our thematic analysis. For example, posts in ED forums referencing the COVID-19 pandemic using terms such as *SARS-CoV-2*, *epidemic*, or *infection* were not included in this study; however, they may contain discussion whose topic or emotion differs from those included in this investigation. Further, posts including only misspelled variations of keywords were not considered. Similarly, only three ED-related subreddits were included in this analysis (ie, r/EatingDisorders, r/AnorexiaNervosa and r/BingeEatingDisorder). It is possible that subreddits without a specific focus on disordered eating behavior may host conversation detailing the influence of the COVID-19 pandemic on eating disorder symptomatology. For example, subreddits such as r/AskReddit and r/AskDocs provide a platform for general health inquiries and patient conversation with nonexperts in medical matters. Individuals posting in such discussion forums may differ from those using ED-specific forums, especially in terms of ED awareness, diagnostic history, and treatment schedule. Future investigation would benefit from a broader investigation of disordered eating behavior during the COVID-19 pandemic among Reddit users posting in non-ED discussion forums. Third, we were not able to confirm the diagnostic history of the Reddit users. Additionally, the demographic characteristics of users included in this investigation is not clear; the users may not be representative of the diverse spectrum of individuals with ED registered to the Reddit community.

### Conclusions

For the majority of individuals participating in ED discussions on Reddit, the COVID-19 pandemic has contributed to increased psychiatric distress, disordered eating behavior, and negative body image. In general, Reddit discussion forums have provided a therapeutic community for individuals to share their experiences, seek advice, and provide support for peers with ED. Future research is needed to further assess the impact of COVID-19 on disordered eating behavior and to evaluate the role of social media discussion forums in mental health treatment, especially during periods of limited treatment access, when the already difficult challenge of reaching individuals with ED is exacerbated.

## Appendix

Multimedia Appendix 1 Table S1. Primary and secondary themes of the Reddit posts with descriptions and examples.

## Abbreviations

ED: eating disorder
