# Supplementary material for: Impact of the COVID-19 Pandemic on Disordered Eating Behavior: Qualitative Analysis of Social Media Posts
Source: JMIR Ment Health. 2021 Jan 27;8(1):e26011. doi: 10.2196/26011 (PMC7842857; doi:10.2196/26011)
Supplement: Multimedia Appendix 1 [file mental_v8i1e26011_app1.docx]

Table S1: Primary and secondary themes of the posts.

| Primary theme | Secondary themes | Description | Examples |
| --- | --- | --- | --- |
| 1. Change in ED^a^ symptoms | Increased ED symptomatology | User indicates the development or significant increase in ED-related thoughts or behaviors due to the COVID-19 pandemic or quarantine. Examples of ED thoughts and behaviors include anxiety related to food or weight, restriction, bingeing, purging, overexercising, and negative self-talk. | “With the recent quarantine, I am unable to work out the same... just running and doing as much as I can. I’ve found my body changing in ways I am very uncomfortable with. I’m waking up daily weighing myself, logging my food, and checking my Fitbit constantly. I recently started staring in the mirror more and despising the person who looks back. I know this isn’t normal. I know throwing up occasionally [because] I hate the way food makes my body feel isn’t normal.” |
|  | Decreased ED symptomatology | User indicates a decrease in ED-related thoughts or behaviors due to the COVID-19 pandemic or quarantine. | “I’ve been bulimic for a pretty small amount of time, and I consider myself lucky. It started in September 2019, it’s currently May 2020. During this time, bulimia consumed my life. I couldn’t go 10 minutes without thinking about my body or calorie intake. I would vomit 2/4 times per day. I’ve been waiting to have a long period of time to recover, and I took quarantine as an opportunity. I got down to only vomiting once a day, then every other day, where I am now.” |
|  | Negative body image | User expresses body dysmorphia, body-related insecurities, or negative feelings toward their body. This encompasses discontent with weight gain, fear of weight gain, and body avoidance behavior. | “I stepped on the scale and it said 195.8 lbs. I was 184.8 on march 16. My weight scale was even surprised of how much weight I gained. I can see a noticeable difference in the mirror. My love handles are bigger, chest is less defined and has more fat, and the biggest difference is my stomach.” |
| 2. Exercise routine | Change in exercise behavior | Quarantine led to a change in exercise behavior (ie, overexercising, inability to exercise). For some users, COVID-19–related restrictions negatively impacted motivation to exercise, contributing to negative emotions such as guilt and anxiety. | “I don’t know why but for the past month or two since covid I’ve been running outside or nowadays on the treadmill for about an hour minimum, some days more or less but around 5/8 miles 3/4 times a week. In addition, I’ve been doing strength training and HIIT^b^ [*sic*] 4 times as well, but I’ve realized how much my bulimia is making me run more longer and I feel better after it.” |
|  | Exercise facilities closed/inaccessible | User explicitly mentions the inability to continue normal exercise activity due to changes in facility accessibility. | “My gym closed down a couple days ago because of the coronavirus I'm getting really anxious right now, I'm starting to eat right and everything but I need the gym in my life.” |
| 3. Impact of quarantine on daily life | Change in routine/environment | In terms of environment, the user either (1) moved to or returned to a different environment due to quarantine or (2) experienced changes within their environment, such as roommates coming or leaving. In terms of routine, the user’s daily routine has been altered due to quarantine. Changes in routine include any shift in daily routine due to quarantine. This may include job loss or school closures, or simply the inability to engage in normal activities. Travel plans may have been cancelled or altered. Additionally, many users mention boredom due to the lack of normal activities during quarantine. | “I'm a senior in college, and I had to come back to my parents' home [because] of COVID. I struggle with restricting and purging. My parents don't know about ED, but will make harsh comments and jokes about weight/calories. It's to the point where I have so much anxiety about eating in front of them that I restrict unless I exercise or am alone. I'm so anxious [because] there's a possibility that I'll be stuck at home for months until I move for grad school, and living inside my head has been like hell.” |
|  | Food hoarding or shortages | The user discusses the urge or need to stock up on food due to the COVID-19 pandemic or grocery/food shortages resulting from the COVID-19 pandemic. | “I don’t know where to go to get water and veggies. Where do I even go??? I’m too scared to eat anything else other than my regular veggies/meats. All the chicken was gone too. I’m frustrated from it all and I feel like I’m on the verge of tears. What am I supposed to eat??? All of this is just going to make me relapse again.” |
|  | Navigating triggering relationships | During quarantine, user experiences interactions with an individual who negatively contributes to ED thoughts or behaviors. | “I moved back in with my family after the quarantine started because one of my roommates still goes to work... The stress of her lately has left me binging late at night. After dinner tonight, I said I was so full. She said ‘You need to go back to starving yourself to shed that weight.’ I was mortified.” |
| 4. Emotional well-being | Negative affect | User describes negative emotions related to current events and associated disordered eating behavior. Commonly described emotions include fear or anxiety, loneliness and isolation, anger or frustration, guilt or shame, and hopelessness or depression. | “I haven’t binged for a long time now but I’m feeling like this quarantine thing is getting to me. I’m feeling lonely and doesn’t know how to go on with my life.”  “I don't know how to stay motivated anymore. I feel like I'm going to fight this every day for the rest of my life and lose.”  “Needing to binge and not being able to is seriously terrible. It’s all I can think about. I’m irritable. Not even irritable, I’m straight up angry and sad.” |
| 5. Help-seeking behavior | Willingness to recover | User indicates motivation to focus on recovery or to decrease disordered thoughts or behaviors during the pandemic/quarantine. The user may be celebrating a victory in the recovery process, such as a positive weight change or a reduction in bingeing, purging, or restricting. | “So today I decided to start fresh. Focus on WHAT I CAN DO by practicing portion control and eating HEALTHY foods.”  “I'm on day three without bingeing (longest since quarantine began), and I feel proud and in control.” |
|  | Currently receiving treatment | Any mention of currently receiving professional treatment | “I have a therapist who I talk to regularly…” |
|  | Unable to receive treatment | User indicates that the current circumstances (1) has led to disruption in their current treatment schedule or (2) has left the user unable to receive professional treatment. In some circumstances, the user may be able to seek help in one domain, but not another (ie, able to use telehealth services, but not group therapy). | “COVID/19 cancelled all group sessions until further notice and I feel so isolated and alone with my thoughts.” |
|  | Requesting advice or accountability from other Reddit users | User explicitly seeks the advice or support of other users. The user may also be in search of an “accountability buddy” to assist them in reducing their ED behavior. | “I guess I’m just looking for other people who have been struggling with relapse in quarantine and also need support because I feel so alone in this.” |
|  | Words of encouragement | User posts an encouraging message directed at themselves or other Reddit users. | “You will get through this, and it will get better. We’ve got this!” |
|  | Seeking help on behalf of another individual | User posts to (1) obtain advice related to supporting those with an ED or (2) has noticed disordered behavior in a friend or family member during quarantine. Posts meeting this criterion were not subsequently assessed for changes in ED behavior or emotionality. | “My girlfriend has started purging and just told me that she hasn’t eaten since Tuesday… What can I do to help her?” |
| 6. Associated risks and health outcomes | Substance use behavior | User indicates substance use behavior, including prescribed and nonprescribed use of psychotropic medications. | “But now since quarantine began like 40 days ago I have sometimes been fasting, restricted, restarted smoking cigarettes and more cigarettes than I had ever on such an amount of time...” |
|  | Adverse health outcomes | User indicates adverse physical health outcomes associated with their ED, including physical pain following ED behavior. | “I feel physically uncomfortable and disconnected from my body, especially when going on walks or trying to run. My body feels tense and bloated and just hurts.” |

^a^ED: eating disorder.

^b^HIIT: high intensity interval training.
